# Development and validation of a simple-to-use nomogram for predicting severe scrub typhus in children

**DOI:** 10.1371/journal.pntd.0013090

**Published:** 2025-05-08

**Authors:** Yonghan Luo, Yan Guo, Yanchun Wang, Xiaotao Yang

**Affiliations:** 1 Second Department of Infectious Disease, Yunnan Key Specialty of Pediatric Infection (Training and Education Program)/Kunming Key Specialty of Pediatric Infection, Kunming Children’s Hospital, Kunming, Yunnan, China; 2 Faculty of Life Science and Technology, Kunming University of Science and Technology, Kunming, Yunnan, China; 3 Department of Reproductive Gynecology, The First People’s Hospital of Yunnan Province, Kunming, Yunnan, China; Universidade do Estado do Rio de Janeiro, BRAZIL

## Abstract

**Objective:**

This study aimed to develop and validate a simple-to-use nomogram for predicting severe scrub typhus (ST) in children.

**Methods:**

A retrospective study of 256 patients with ST was performed at the Kunming Children’s Hospital from January 2015 to November 2022. ALL patients were divided into a common and severe group based on the severity of the disease. A least absolute shrinkage and selection operator (LASSO) regression model was used to identify the optimal predictors, and the predictive nomogram was plotted by multivariable logistic regression. The nomogram was assessed by calibration, discrimination, and clinical utility.

**Results:**

LASSO regression analysis identified that hemoglobin count (Hb), platelet count (PLT), lactate dehydrogenase (LDH), blood urea nitrogen (BUN), creatine kinase isoenzyme MB(CK-MB) and hypoproteinemia were the optimal predictors for severe ST. The nomogram was plotted by the six predictors. The area under the receiver operating characteristic (ROC) curve of the nomogram was 0.870(95% CI = 0.812 ~ 0.928) in training set and 0.839(95% CI = 0.712 ~ 0.967) in validation set. The calibration curve demonstrated that the nomogram was well-fitted, and the decision curve analysis (DCA) showed that the nomogram was clinically beneficial.

**Conclusions:**

This study developed and validated a simple‐to‐use nomogram for predicting severe ST in children based on six predictors including Hb, PLT, LDH, BUN, CK-MB and hypoproteinemia, demonstrating excellent predictive accuracy for the data, though external and prospective validation is required to assess its potential clinical utility.

## Author summary

Scrub typhus (ST) is a serious infectious disease caused by *Orientia tsutsugamushi* transmitted through mite bites. It is particularly dangerous for children, and predicting its severity is critical for timely intervention. This study aimed to develop a simple, user-friendly tool to predict severe ST in children. Through a retrospective analysis of clinical data from 256 ST patients, we identified six critical factors—hemoglobin count (Hb), platelet count (PLT), lactate dehydrogenase (LDH), blood urea nitrogen (BUN), creatine kinase isoenzyme MB(CK-MB) and hypoproteinemia—that can predict disease severity. Based on these six factors, we developed a nomogram tool to assist healthcare providers in quickly assessing the risk of severe ST in children. The validation of the nomogram demonstrated its accuracy and utility in clinical decision-making. Our findings suggest that this nomogram could serve as a valuable tool in pediatric healthcare, aiding physicians in identifying children at higher risk for severe ST and making more informed treatment decisions.

## Introduction

Scrub typhus (ST) is a zoonotic illness caused by *Orientia tsutsugamushi* (OT). rats are the main source of infection and trombiculid mites are the transmission vector [1]. larval stage mites (‘chiggers’) bites human tissue and causes skin lesions that eventually form clinically characteristic ulcers or eschar [2]. Pathogens can be distributed to various organs or tissues of the body along lymphatic and blood circulation [3].They infiltrate endothelial cells of different organs from the lymph nodes, with these endothelial cells located in the heart, lungs, brain, liver, and kidneys [4]. Vascular endothelial damage and excessive inflammatory responses may result in systemic poisoning symptoms, damage lesions of corresponding organs and tissues, and even multiple organ dysfunction syndrome (MODS) or death [5]. A meta-analysis [6] of 89 studies indicated that the overall case-fatality rate for ST was approximately 12.7%, but the overall variability was large (0–70%) and the mortality rate could be as high as 30% in severely ill patients [7]^.^ A recent meta-analysis [8] from articles across 29 countries/regions showed that Mainland China had the highest number of cases, while South Korea and Thailand reported the highest incidence rates. The median mortality rates were 5.00% among hospital inpatients, 6.70% among patients with unspecified admission status, and 2.17% among outpatients. Therefore, early identification of the risk of severe ST and timely treatment are very important. Studies [7] have shown that rapid diagnosis and prompt treatment can shorten the disease course and achieve a good prognosis, especially in younger patients. Babu [9] et al. showed that indicators such as the presence of peripheral edema, decreased hemoglobin(Hb), longer duration of illness, absence of eschar and other indicators may be predictors of severe ST. But the area under the curve of these predictors was not high. Panda et al [10]. identified hepatomegaly, rashes, and lymphadenopathy as significant clinical indicators for predicting the severity of the condition. Similarly, Guan et al [11]. found that the presence of peripheral edema and decreased hemoglobin levels were the most critical predictors of severe illness in pediatric patients. However, in studies of pediatric ST, it is often clinically impractical to rely on a single laboratory indicator to predict disease severity.In addition, previous studies [12,13] tend to focus on adults, and there is still a lack of research on the predicting model of severe ST in children. Considering the above reasons, we developed a nomogram to predict the probability of severe ST by combining the clinical and laboratory characteristics of children with ST in our hospital. The objective is to provide scientific a basis for early detection, diagnosis and treatment, to reduce the morbidity and mortality of severe ST.

## Materials and methods

### Ethics statement

Although informed consent was waived due to the retrospective nature of the study, this study was approved by the Ethics Review Committee of Kunming Children’s Hospital. Furthermore, we ensured that all patient data were anonymized to maintain confidentiality and privacy, in accordance with ethical standards. This study was carried out in accordance with the ethical standards of the Declaration of Helsinki.

### Study population and general data

The clinical data of 256 cases with ST at the Kunming Children’s Hospital from January 2015 to November 2022 were analyzed retrospectively. This study was approved by the Ethics Committee of the Kunming Children’s Hospital. The requirement of obtaining informed consent was waived due to no clinical trials or randomized controlled trials.

Inclusion criteria: (1) hospitalized patients under 18 years old; (2) in accordance with one of three diagnostic criteria of pediatric ST of Pediatric Infectious Diseases (5 version) [14]: 1) patients who had a history of field activities in an epidemic region/ season within 3 weeks prior to illness onset and presented sudden high fever with characteristic eschar or ulcer, rash, lymphadenopathy and other typical clinical manifestations can be clinically diagnosed ST.2) At least one laboratory test is positive: specific IgM antibody positive or double serum antibody titer increased by more than 4 times (Weil-Felix test) or pathogen detected by polymerase chain reaction (PCR). 3) ST was highly suspected but could not be diagnosed, and fever was reduced within 48 hours after diagnostic treatment with azithromycin or doxycycline. Exclusion criteria: (1) had underlying disease, such as congenital heart disease, hematological malignant tumor or immunodeficiency;(2) serious lack of clinical data, meaning that the available clinical information for an individual patient is insufficient to determine whether they belong to the severe group or the common group.

### Definition of disease grouping

The diagnostic criteria of severe ST in this study were referred to the study of PARK et al [15]

Central nervous system: change of consciousness, convulsions, cerebral hemorrhage, or cerebral infarction; (2) respiratory system: chest X-ray or CT showed bilateral lung infiltration, or oxygenation index ≤ 250mmHg, or increased respiratory rate (RR) (RR > 70/ min in infants, RR > 50/ min in children over 1 year old), or required mechanical ventilation; (3) circulation system: myocarditis, myocardial ischemia, or new arrhythmia. (4) urinary system: serum creatinine ≥ 176umol/L; (5) septic shock: systolic blood pressure decreased; (age 1–12 months old) < 70 mmHg, (age 1–10 years old) <[70 + (2 age)] mmHg; (age ≥ 10 years old) < 90mmHg;(6) gastrointestinal bleeding (without peptic ulcer basis); (7) death. Cases can be included in the severe group in accordance with any of the above, the rest were included in the common group.

### Data sources

Data collected from hospital electronic medical records include demographic characteristics(age, sex, season of onset), Symptoms(cough, fever, a clear history of field activities before onset, vomiting, abdominal pain, headache, The duration of fever before hospitalization), signs(ulcer or eschar, lymphadenopathy, hepatomegaly, splenomegaly, edema), complications(pneumonia, liver function damage, hypoproteinemia, electrolyte disturbance, meningoencephalitis or encephalitis, coagulation abnormalities, renal dysfunction, gastrointestinal bleeding, serous effusion, myocardial damage, hemophagocytic syndrome, shock, MODS, death), laboratory examination(WBC, EO, Hb, PLT, CRP, PCT, ALT, AST, TBil, SCr, BUN,UA,CK-MB,LDH,PT,APTT,FIB), treatment(Course of disease before doxycycline or/azithromycin used), outcome(Length of stay, improvement and discharge/death) (Table 1).

**Table 1:** The clinical characteristic and laboratory tests in common group and severe group.

	Common group(n = 204)	Severe group(n = 52)	ALL patients(n = 256)	P
**Clinical characteristic**				
Sex (male) [n (%)]	112(54.9)	27(51.9)	139(54.5)	0.7
Age, M (IQR), years	5.71 ± 3.62	4.38 ± 3.16	5.44 ± 3.57	0.111
**The season of onset**				
Spring [n (%)]	7(3.4)	2(3.8)	9(3.5)	0.987
Summer [n (%)]	123(60.3)	32(61.5)	155(60.5)	
Autumn [n (%)]	64(31.4)	16(30.8)	80(31.3)	
Winter [n (%)]	10(4.9)	2(3.8)	12(4.7)	
**Symptoms**				
Cough [n (%)]	101(49.5)	29(55.8)	130(50.8)	0.42
Fever [n (%)]	204(100)	52(100)	256(100)	–
A clear history of field activities before onset [n (%)]	113(55.4)	12(23.1)	125(48.8)	<0.001
Vomiting [n (%)]	33(16.2)	11(21.2)	44(17.2)	0.396
Abdominal pain [n (%)]	43(21.1)	12(23.1)	55(21.5)	0.754
Headache [n (%)]	43(21.1)	5(9.6)	48(18.8)	0.059
The duration of fever before hospitalization, M (IQR), days	7.00(4.00)	8.00(4.00)	7.00(4.00)	0.609
**Physical signs**				
Ulcer or eschar [n (%)]	172(84.3)	41(**78**.8)	213(83.2)	0.346
Lymphadenopathy [n (%)]	104(51.0)	21(40.4)	125(48.8)	0.172
Hepatomegaly [n (%)]	89(43.6)	35(67.3)	124(48.4)	0.002
Splenomegaly [n (%)]	75(36.8)	22(42.3)	97(37.9)	0.462
Edema [n (%)]	56(27.5)	24(46.2)	80(31.3)	0.009
**Complications**				
Pneumonia [n (%)]	53(26.0)	29(55.8)	82(32.0)	<0.001
Liver function damage [n (%)]	100(49.0)	36(14.1)	136(53.1%)	0.009
Hypoproteinemia [n (%)]	58(28.4)	32(61.5)	90(35.2)	<0.001
Electrolyte disturbance [n (%)]	34(16.7)	20(38.5)	54(21.1)	0.001
Meningoencephalitis or encephalitis [n (%)]	21(10.3)	10(19.2)	31(12.1)	0.078
Coagulation abnormalities [n (%)]	3(1.5)	3(5.8)	6(2.3)	0.188
Renal dysfunction [n (%)]	0(0.0)	2(3.8)	2(0.8)	0.041
Gastrointestinal bleeding [n (%)]	1(0.5)	3(5.8)	4(1.6)	0.027
Serous effusion [n (%)]	5(2.5)	8(15.4)	13(5.1)	0.001
Myocardial damage [n (%)]	5(2.0)	3(5.8)	8(3.1)	0.435
Hemophagocytic syndrome [n (%)]	6(2.9)	22(42.3)	28(10.9)	0.001
Shock [n (%)]	1(0.5)	3(5.8)	4(1.6)	0.027
MODS [n (%)]	16(7.8)	20(38.5)	36(14.1)	<0.001
**Treatment**				
Course of disease before doxycycline/Azithromycin used, M (IQR), days	8.00(4.00)	9.00(4.8)	7.00(4.00)	0.015
**Outcome**				
Length of stay,M (IQR), days	7.00(2.00)	12.5(6.80)	7.00(3.00)	<0.001
Death [n (%)]	0(0.0)	2(3.8)	2(0.8)	0.041
**Laboratory data**				
WBC, M (IQR), × 10^9^/L	8.62(6.51)	9.06(7.30)	8.74(6.85)	0.110
EO, M (IQR), × 10^9^/L	0.01(0.02)	0.00(0.01)	0.00(0.02)	0.028
Hb, M (IQR), × g/L	110.00(21.00)	97.00(21.00)	108.00(22.00)	<0.001
PLT, M (IQR), × 10^9^/L	106.50(113)	35.00(53.00)	93.00(113.75)	<0.001
CRP, M (IQR), mg/L	35.33(54.17)	57.98(68.15)	37.41(53.83)	0.005
PCT, M (IQR), ng/ml	2.97(4.01)	4.58(3.77)	3.14(3.76)	0.004
ALT, M (IQR), U/L	65.50(85.65)	97.50(125.40)	70.50(93.84)	0.007
AST, M (IQR), U/L	77.05(89.30)	221.00(283.98)	90.50(174.73)	<0.001
TBil, M (IQR), umol/L	8.45(4.88)	10.85(17.90)	8.80(5.83)	0.045
SCr, M (IQR), µmol/L	28.90(12.93)	29.51(24.73)	28.95(14.67)	0.097
BUN, M (IQR), mmol/L	3.61(2.02)	4.95(5.87)	3.80(2.06)	<0.001
UA, M (IQR), µmol/L	240.50(99.50)	284.50(153.88)	263.4(98.01)	0.270
CK-MB, M (IQR), U/L	20.00(11.00)	29.00(28.25)	21.00(13.00)	<0.001
LDH, M (IQR), U/L	570.50(282.80)	830.50(720.00)	609.50(324.75)	0.002
PT, M (IQR), s	13.50(6.14)	17.00(8.50)	14.00(5.94)	<0.001
APTT, M (IQR), s	37.95(11.95)	42.65(15.75)	39.00(13.35)	0.001
FIB, M (IQR), g/L	2.40(3.08)	3.37(3.86)	2.50(3.00)	0.018
Agglutination titer ≥ 1:160 of weil-Felixtest [n (%)]	84(41.2)	21(40.4)	105(41.0)	0.917

*WBC (white blood cell), EOS (eosinophils), Hb(hemoglobin), PLT (platelet), CRP (C -reactive protein), PCT (procalcitonin), ALT (alanine aminotransferase), AST (alanine aminotransferase), TBil(total bilirubin), SCr(serum creatinine), BUN(blood urea nitrogen), UA (uric acid), CK-MB(Creatine kinase isoenzyme), (LDH)lactate dehydrogenase, PT(Prothrombin time), APTT(activated partial thromboplastin time), FIB(fibrinogen)*

### Definitions

Pneumonia is defined as presence of fever or acute respiratory symptoms accompanied by evidence of parenchymal infiltrates on a chest radiograph [16]. Liver function damage: ALT or AST level ≥ upper limit of normal. Hypoalbuminemia is defined as serum ALB lower than 30g/L [17]. Meningoencephalitis or encephalitis are defined as clinical symptoms such as confusion of consciousness, disorientation and hallucination or accompanied by leukocytosis in cerebrospinal fluid. Electrolyte disorder is defined as deviating from normal levels of sodium, potassium, chlorine, magnesium and calcium. Kidney disfunction is defined as a loss of excretory kidney function, clinically characterized by decreased urine volume and increased creatinine level [18]. The abnormality of blood coagulation is mainly manifested by prolonged PT and APTT. Shock is defined as severe circulatory failure that requires vasopressor or inotropic infusion [19].

### Statistical analysis

The normally distributed continuous variables were compared by t-test and expressed as X ±standard deviation, while the non-normally distributed continuous variables were compared by the Mann-Whitney test and expressed as the median of the inter-quartile ranges (IQR). Categorical variables were analyzed by χ2 test or Fisher’s exact test and expressed as numbers (n) and percentages (%). The differences in laboratory tests between the two groups were compared visually. All cases were divided into a training (70% of the data) and a validating (30% of the data) set by using the “Sample ()” function in R software. The optimal predictors were selected by the least absolute shrinkage and selection operator (LASSO) regression method. The predictors picked were used to construct a prediction model, which was displayed with a nomogram. The discrimination of the nomogram was evaluated by the area under receiver operating characteristic (AUC). The accuracy of the model was accessed by a calibration curve. The clinical utility of the nomogram was evaluated using decision curve analysis (DCA). p < 0.05 was considered to be statistically significant. All statistical analysis was performed with R software (version 3.60).

## Results

### Clinical characteristics

A total of 256 cases (139 males and 117 females) with ST were included in this study. The average age was 5.44 ± 3.57 years old. The most common seasons of ST in this study are summer (60.5%) and autumn (31.3%). All cases had fever, and 213 (83.2%) cases had clear signs of ulcer or eschar. All cases were treated with doxycycline (234 cases, 91.4%) or azithromycin (22 cases, 8.6%) immediately after being diagnosed as ST. Eventually, 254 (99.2%) cases were discharged after improvement or recovery, and only 2 (0.8%) cases died.

**Fig 1:**
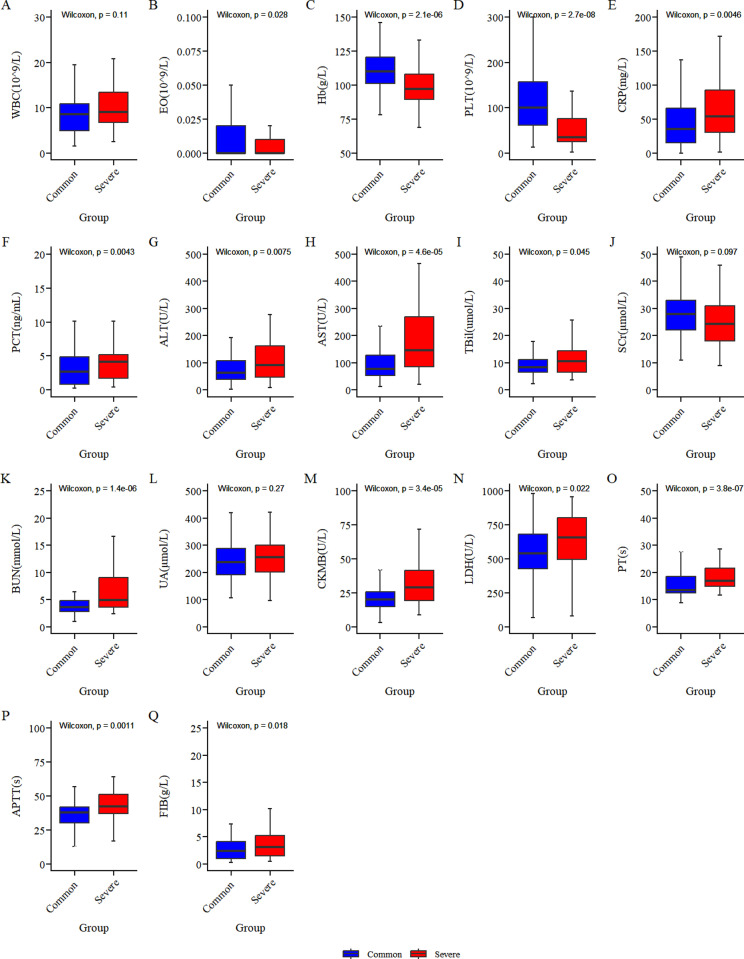
A bar chart of the different laboratory indicators in common group and severe group.

Univariate analysis showed that there was no significant difference in the incidence of cough, fever, vomiting, abdominal pain, headache, ulcer or eschar, lymphadenopathy, splenomegaly, reactive encephalitis, coagulation abnormalities, myocardial damage, and an agglutination titer ≥ 1:160 of the Weil-Felix test between the two groups (P > 0.05). There was no significant difference in the duration of fever before hospitalization, WBC, and SCr between the two groups (P > 0.05). There were significant differences in the reporting of a clear history of field activities before onset, hepatomegaly, edema, pneumonia, liver function damage, hypoproteinemia, electrolyte disturbance, renal dysfunction, gastrointestinal bleeding, serous effusion, hemophagocytic syndrome, shock, MODS, death, and course of disease before doxycycline or azithromycin was used between the two groups. There were significant differences in EO, Hb, PLT, CRP, PCT, ALT, AST, TBil, BUN, UA, CK-MB, LDH, PT, APTT, FIB, course of disease before doxycycline was used, and length of stay between the two groups. (See Table 1) A bar chart of the different laboratory indicators of the two groups can be seen in Fig 1.

### Selecting predictors by the LASSO regression method in the training set

**Fig 2:**
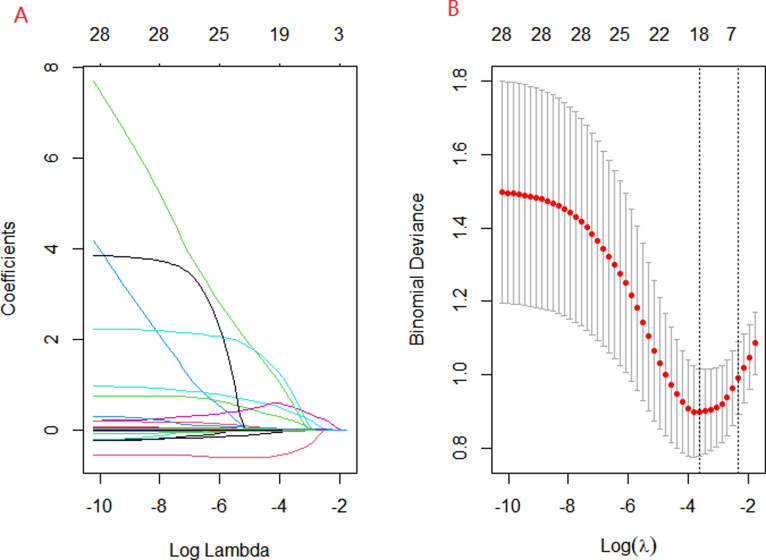
Predictors’ selection using LASSO regression method. **(A)** LASSO coefficient profiles of the 28 variables. The coefficient profile plot was produced against the log (λ) sequence. **(B)** The best penalty coefficient lambda was selected using a tenfold cross-validation and minimization criterion. By verifying the optimal parameter (λ) in the LASSO model, the binomial deviance curve was plotted versus log(λ) and dotted vertical lines were drawn based on 1 standard error criteria. 6 variables with nonzero coefficients were selected by optimal lambda.

A univariate analysis showed that there were statistical differences in 33 variables between the severe group and the common group. However, the purpose of this study is to establish an early predictive model. So, two outcome variables, including death and length of stay, as well as three complication variables, including MODS, shock, and hemophagocytic syndrome, are excluded. Finally, twenty-eight variables with statistical differences were analyzed by using the LASSO regression model in the training set (Fig 2A). The LASSO model is a logistic regression model that can transform the penalty function into an absolute value while compressing the regression coefficient to select predictors. It can help reduce over-fitting and multicollinearity in a large number of variables selected during modeling. The one standard error of the minimum was determined by using cross-validation in LASSO logistic regression, which suggested that Hb, PLT, LDH, BUN, CK-MB, and hypoproteinemia were optimal predictors for severe ST (Fig 2B).

### Development of the nomogram

**Fig 3:**
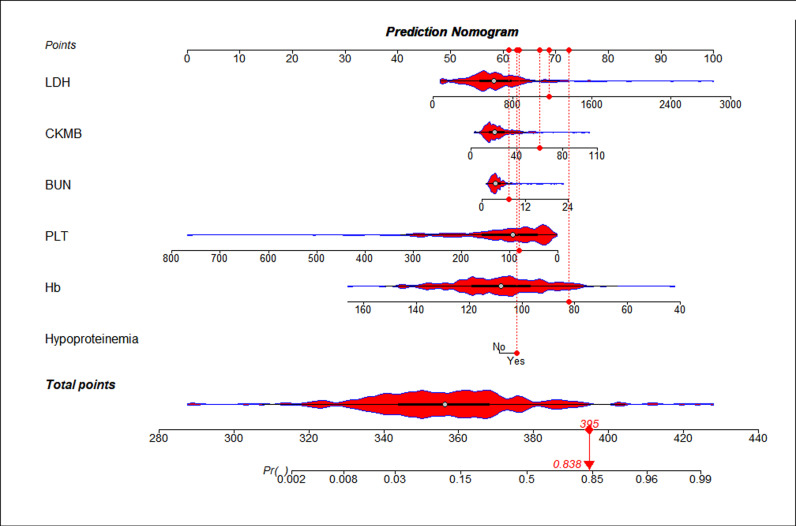
Nomogram was plotted based on six optimal predictors for severe scrub typhus. According to whether the cases had hypoproteinemia, BUN, CK-MB, LDH, Hb and PLT, we can get a point on top lines. then add each point to obtain a total points and project it vertically on the bottom axis to obtain a predicted value of severe scrub typhus*.*

A predictive nomogram containing six independent predictors (Hb, PLT, LDH, BUN, CK-MB and hypoproteinemia) was established by logistic regression (Fig 3). The logistic regression equation was logit(P)= (2.095) -0.043X_Hb_-0.01X _PLT_ + 0.01X _LDH_ + 0.053X_BUN _+ 0.025X_CK-MB_ + 0.631X_hypoproteinemia_. The cut-off point of the probability used to declare a case of ST as severe and non-severe is 0.671. A patient with ST who had hypoproteinemia, for example, had a Hb of 82 g/L, a PLT of 80 10^9^/L, a LDH of 1170 U/L, a CKMB of 60 U/l and a BUN of 7.45mmol/L, totaling 395 (62 + 74 + 63 + 68 + 67 + 61). The predicted risk is 0.838. (Fig 3)

### Validation of the nomogram

**Fig 4:**
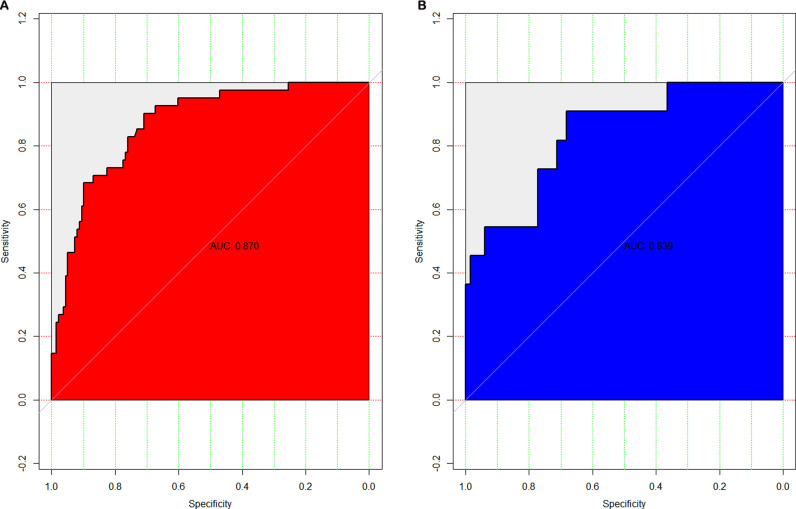
ROC curve of predictive nomogram. The red represents in the training set and the blue represents the testing set.

**Fig 5:**
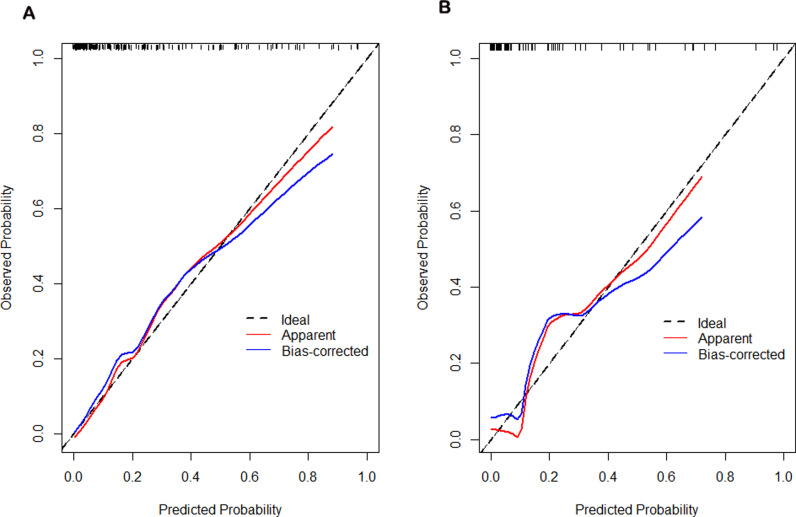
Calibration plots of the nomogram in the training set(A) and testing set(B).

1000 bootstrap analyses were performed for internal validation of the nomogram; the AUC of the nomogram was 0.870 (95% CI = 0.812–0.928) in the training set and 0.839 (95% CI = 0.712–0.967) in the testing set (Fig 4), with good discrimination ability. In the training set, the model’s sensitivity was 0.667 (95% CI: 0.488–0.808), specificity was 0.859 (95% CI: 0.794–0.906), positive predictive value was 0.488 (95% CI: 0.343–0.635), and negative predictive value was 0.928 (95% CI: 0.872–0.960). In the validation set, sensitivity was 0.800 (95% CI: 0.376–0.964), specificity was 0.903 (95% CI: 0.813–0.952), positive predictive value was 0.364 (95% CI: 0.152–0.646), and negative predictive value was 0.985 (95% CI: 0.919–0.997).The calibration curves and Hosmer-Lemeshow test showed good probability consistency between the predicted and actual probabilities in the training(P = 0.75) set and validating (P = 0.40) set (Fig 5).

### Clinical utility of the nomogram

**Fig 6:**
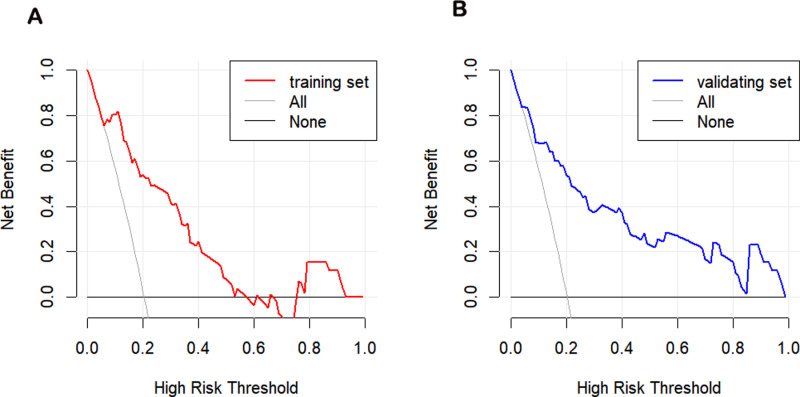
Decision curve analysis (DCA) for the predictive model. the training set(A). the validating set (B).

**Fig 7:**
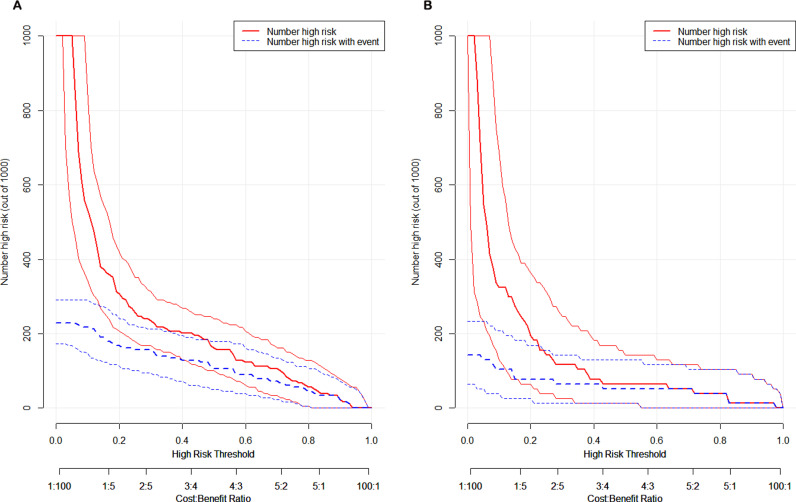
Clinical impact curve for the predictive model. (**A)** the training set. (**B**) the validating set.

The clinical utility of the nomogram was analyzed using the DCA curve. Fig 6 showed that the net benefit was > 0 when the high-risk threshold was 0.1 ~ 0.9 in the training set and 0.1 ~ 1.0 in the validating set, which suggested that the nomogram has good clinical utility in predicting severe ST in children. When the high-risk threshold was greater than 0.4 and 1,000 patients were used for prediction, the clinical impact curve analysis revealed that the predicted number of patients was close to the actual number of patients (Fig 7).

## Discussion

In this study, we developed and validated a simple-to-use nomogram for predicting severe ST in children. To our knowledge, this is the first study on a predictive model of severe ST in children worldwide. Eventually, twenty-eight variables were reduced to six optimal predictors including Hb, PLT, LDH, BUN, CK-MB and hypoproteinemia. Validation of the nomogram showed good accuracy and discrimination. Besides, the predictive nomogram demonstrated good clinical utility, suggesting that it could assist physicians in identifying markers associated with severe ST in children, even if these markers do not necessarily explain the underlying mechanisms of the disease.

The main endemic area of ST is in a “ tsutsugamushi triangle”, from Japan in the east, Australia in the south, Pakistan in the west, and Russia Far Eastin the north [20–22].Since 2006, the incidence of ST in China has been increasing year by year, with an annual growth rate of up to 32%, showing a trend of spreading from the coastal cities in southeast China to the west and north [23]. ST has also spread worldwide from the traditional triangular belt to regions including West Asia, Africa and South America. Yunnan Province, where the cases were collected in this study, is located in this triangle, one of the highest incidences of ST in China [23]. In this study, the prevalence of ST was mainly in summer and autumn, and the peak was from June to August, which was consistent with the previous epidemic season of ST in Yunnan Province [24]. The main epidemic season of ST was different in different regions. The higher the latitude was, the later the epidemic peak month was. For example, the epidemic peak in low latitude areas such as Vietnam and southern China mainly concentrated in June and July, while the epidemic peak in high latitude areas such as Japan and South Korea mainly occurred in October and November [23]. This is related to climate. Yunnan Province is located in the subtropical monsoon climate zone, and the summer is warm and humid, which is just conducive to the growth of trombiculid mites.

It is believed that the pathogenic mechanism of ST is complex, involving the innate, cellular and humoral immune systems. ST can cause local or systemic inflammatory reactions, leading to vasculitis and perivascular inflammatory lesions in single or multiple tissues, organs, and systems, resulting in vascular leakage and eventual organ damage. The skin, liver, lungs, kidneys, brain and other organs can be affected [25]. Therefore, we summarized the clinical manifestations and laboratory indicators that may be associated with these multi-organ damage to predict the occurrence of severe ST.

In this study, decreased Hb and PLT were included as predictors of severe ST. This result is consistent with previous studies. guan [11] et al. conducted a retrospective study showed that reduced Hb was the most important factors of severe ST in pediatric patients, with an OR of 13.22 (1.54-113.50). The study by Park et al [15]. concluded that anemia (≤ 10 g/dL) was indicators of current severity. Several studies [26–28] have identified thrombocytopenia as a predictor of ST severity. The mechanism of thrombocytopenia is mainly related to the damage of endothelial cells (EC). Pathogens and postmortem toxins directly cause vascular endothelial damage when flowing through the blood, and PLT are consumed in large quantities after vascular endothelial damage. Direct damage to megakaryocytes also results in thrombocytopenia [29]. In addition, secondary hypersplenism and inhibition of megakaryocytes in bone marrow are also the causes of thrombocytopenia. It is worth mentioning that hemophilia syndrome as one of the complications of severe ST has been reported in many cases [30]. Hemophagocytic syndrome results from uncontrolled activation of inflammatory cytokines and is characterized by the aggregation of activated macrophages and lymphocytes. Tameto [25] reviewed the literature from 2016 to 2017 and found nearly 30 cases of ST complicated with hemophagocytic syndrome, 13 cases of which were children, with a mortality rate of 6.7%. Takami et al [31]. suggested that the function of macrophages is triggered in the early stages of ST. Given the critical severity of the hemophagocytic syndrome, a decrease in PLT and Hb should be of particular concern to clinicians.

At present, many studies [27,32] have confirmed the close relationship between ALB decrease and ST. Similarly, this study further strengthened this conclusion by showing that hypoproteinemia is a predictor of severe ST. The decrease of ALB may be related to the extravasation of ALB caused by vasculitis, the consumption of ALB by acute infection and the influence of liver ALB synthesis. In addition, ALB is also considered to be a negative indicator of acute inflammation [33], and its reduction reflects the intensity of inflammation in the body, which is often related to the severity of the disease. We also found an association between reduced LDH levels and severe ST, which was consistent with previous studies [34]. LDH is an important enzyme in the glycolytic pathway and widely exists in human tissue cells. The concentration of LDH in various tissue cells is 1500 ~ 5000 times that of serum [35]. The systemic inflammatory response induced by ST causes the release of LDH.

Acute kidney injury (AKI) is an underestimated complication of ST and also a predictor of mortality [36]. Previous studies [15] have often used SCr levels as a risk factor. Although SCr levels were found to be statistically different between the severe and non-severe groups in our study, we excluded it after lasso regression analysis and selected elevated BUN as a predictor. It is suggested that BUN as a marker of renal damage in patients with ST should be paid more attention by clinicians.

There are some limitations in this study. First, as this study is a single-center retrospective study in Kunming, it may lead to selection bias. Secondly, some potential predictors of severe ST such as ferritin, cytokines and gene types, were unable to be included in the statistics due to the lack of data in the samples. Finally, the sample size of this study is relatively small and has not been externally verified, so multi-center and large samples are needed to verify the accuracy of the conclusions in the future.

## Conclusion

In this study, a simple-to-use nomogram prediction model for predicting severe scrub typhus in children was developed and verified based on six predictors including Hb, PLT, LDH, BUN, CK-MB and hypoproteinemia, demonstrating excellent predictive accuracy for the data, though external and prospective validation is required to assess its potential clinical utility.

## Supporting information

S1 Dataset The data set analyzed in this study. (XLSX)
